# Supplementary material for: Application of CRISPR/Cas9 System for Efficient Gene Editing in Peanut
Source: Plants (Basel). 2022 May 20;11(10):1361. doi: 10.3390/plants11101361 (PMC9144340; doi:10.3390/plants11101361)
Supplement: Supplementary file 1 [file plants-11-01361-s001.zip › plants-1722736-supplementary.pdf]

Map of vector pDW3872 (dual *Bsa*I cut sites for insertion of the designed gRNA)

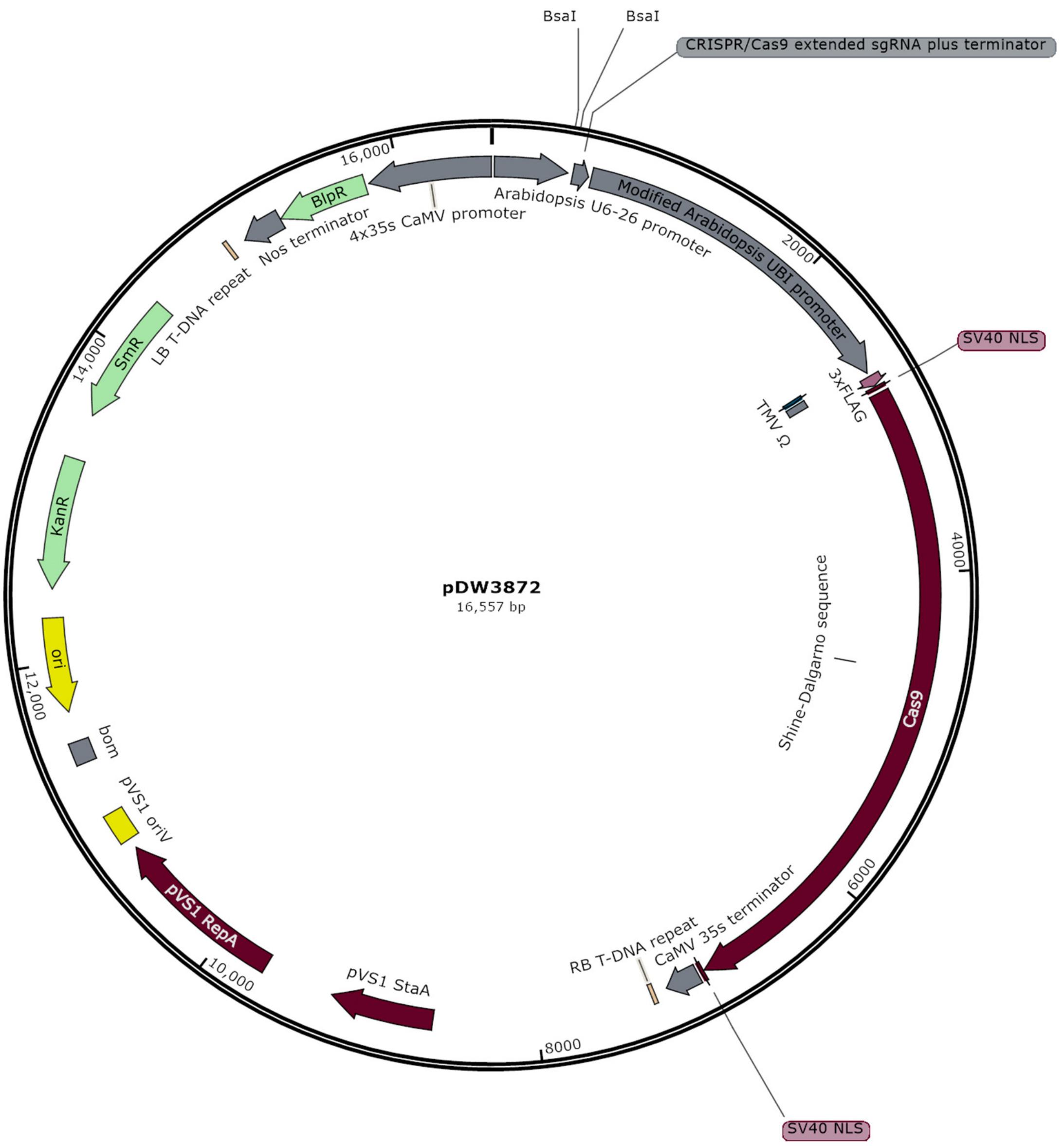

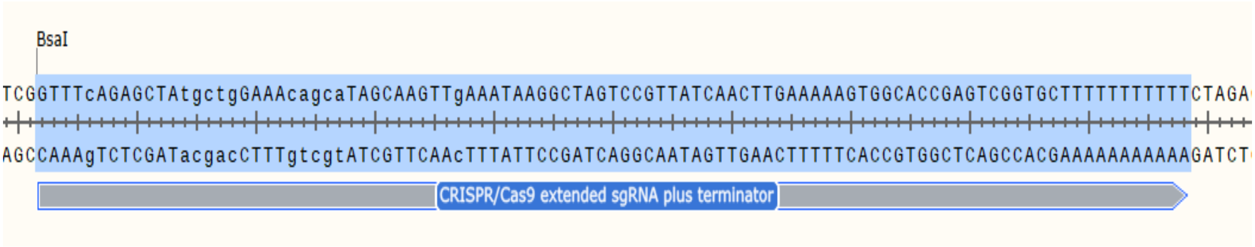

Extended gRNA scaffold plus terminator (492…588 = 97 bp), 40% GC.

Map of vector pDW3877 (dual *Bsa*I cut sites for insertion of the designed gRNA)

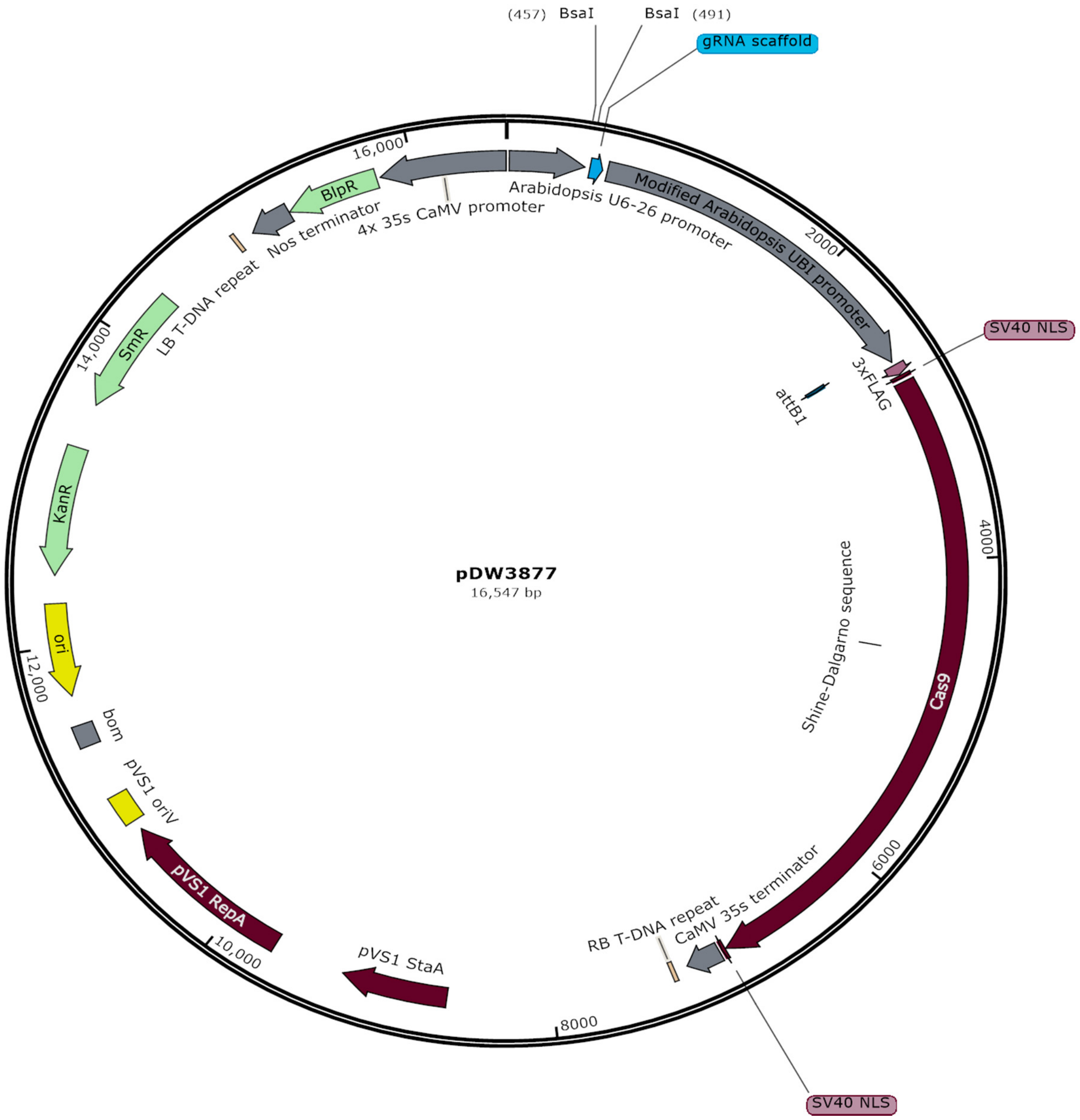

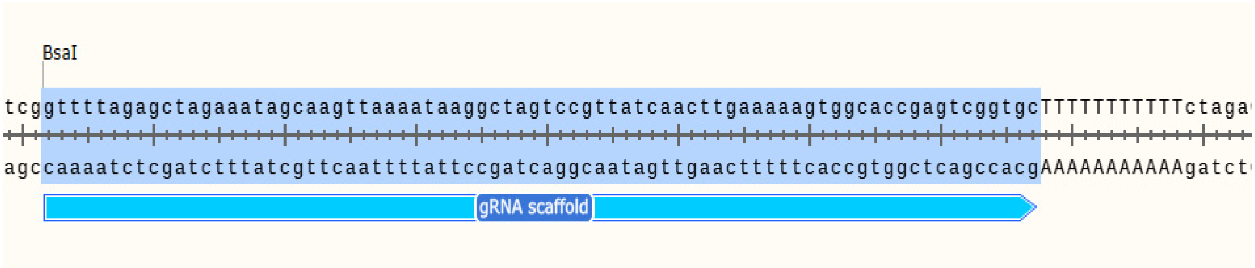

gRNA scaffold (492…567 = 76 bp), 41% GC

Figure S1. Map of two vectors.
